# Supplementary material for: “No Child or Adult Would Ever Probably Choose to Have 16 Teaspoons of Sugar”: A Preliminary Study of Parents’ Responses to Sugary Drink Warning Label Options
Source: Nutrients. 2022 Oct 7;14(19):4173. doi: 10.3390/nu14194173 (PMC9571345; doi:10.3390/nu14194173)
Supplement: Supplementary file 1 [file nutrients-14-04173-s001.zip › nutrients-1946261-supplementary.pdf]

Supplementary material

Figure S1. Graphical representation of study design

**1. Research planning**

- Research question development
- Development of discussion guide and labels to be tested

**2. Data collection:**

12 focus groups of 3-8 participants each

- Initial discussion on sugar-sweetened beverage consumption (own and child's)
- Participants shown each set of labels (see Figure 2 for more detail on label images)
  - Health effects (4 labels)
  - Sugar content + health effects (2 labels)
  - Content and energy information (4 labels)
  - Exercise equivalents (4 labels)
  - High in sugar + teaspoons in pictograms (3 labels)
  - Teaspoons pictograms (4 labels)
- Discussion guide prompted for each label:
  - Initial reactions
  - Taught participants something new?
  - Believable?
  - Made participants stop and think?
  - Considered relevant?
  - Impacted consumption and purchase intentions
  - Caused discomfort over own or children's consumption

**3. Data analysis:**

- Preparation of material for data analysis (transcripts from audio and video recordings)
- Initial inductive and deductive coding within label sets using framework derived from above discussion guide and prior qualitative study with young adults (ref)
- Themes identified across label sets, organised into main themes and sub-themes

**4. Final interpretation and write up:**

- Final consolidation and reporting of themes and sub-themes
- Consideration of practical implications and findings
